# Standardized Herbal Formula PM014 Inhibits Radiation-Induced Pulmonary Inflammation in Mice

**DOI:** 10.1038/srep45001

**Published:** 2017-03-21

**Authors:** Jee-Youn Kim, Dasom Shin, Gihyun Lee, Jin-Mo Kim, Dongwook Kim, Yong-Min An, Byung Rok Yoo, Hanna Chang, Miran Kim, Jaeho Cho, Hyunsu Bae

**Affiliations:** 1 Department of Radiation Oncology, Yonsei University College of Medicine, Seoul, South Korea; 2 Department of Science in Korean Medicine, Graduate School, Kyung Hee University, Seoul, 02447, Republic of Korea; 3 Central Research Institute, Hanlim Pharm. Co. Ltd., 1007 Yoobang Dong, Yongin, Kyounggi Do, Republic of Korea

## Abstract

Radiation therapy is widely used for thoracic cancers. However, it occasionally causes radiation-induced lung injuries, including pneumonitis and fibrosis. Chung-Sang-Bo-Ha-Tang (CSBHT) has been traditionally used to treat chronic pulmonary disease in Korea. PM014, a modified herbal formula derived from CSBHT, contains medicinal herbs of seven species. In our previous studies, PM014 exhibited anti-inflammatory effects in a chronic obstructive pulmonary disease model. In this study, we have evaluated the effects of PM014 on radiation-induced lung inflammation. Mice in the treatment group were orally administered PM014 six times for 2 weeks. Effects of PM014 on radiation pneumonitis were evaluated based on histological findings and differential cell count in bronchoalveolar lavage fluid. PM014 treatment significantly inhibited immune cell recruitment and collagen deposition in lung tissue. Normal lung volume, evaluated by radiological analysis, in PM014-treated mice was higher compared to that in irradiated control mice. PM014-treated mice exhibited significant changes in inspiratory capacity, compliance and tissue damping and elastance. Additionally, PM014 treatment resulted in the downregulation of inflammatory cytokines, chemokines, and fibrosis-related genes and a reduction in the transforming growth factor-β1-positive cell population in lung tissue. Thus, PM014 is a potent therapeutic agent for radiation-induced lung fibrosis and inflammation.

Thoracic radiation therapy is commonly used for treatment of lung and breast cancers as well as various lymphomas^1^,^2^,^3^. However, lung tissues are particularly sensitive to radiation^4^. Consequently, radiation-induced lung injury (RILI), which is classified as early-phase pneumonitis or late-phase pulmonary fibrosis, is a severe, and sometimes lethal, side effect of thoracic radiation therapy. Radiation pneumonitis is characterized by oedema of alveolar spaces, infiltration of inflammatory cells such as macrophages, neutrophils, and fibroblasts into the interstitium, and aggregation of hyaline products. Infiltrated inflammatory cells are activated to release a variety of cytokines, such as transforming growth factor (TGF)-β, interleukin (IL)-1β, tumour necrosis factor (TNF)-α, chemokine ligand (CCL)-2, CCL3, and platelet-derived growth factor (PDGF)^5^,^6^. Transforming growth factor-β, the most extensively investigated radiation-induced cytokine, plays a key role in mediation of tissue response involved in the progress of pneumonitis^7^,^8^. Therefore, this inflammatory cytokine could be an effective inhibitory target for the prevention of radiation pneumonitis.

Herbal medicine has been used for centuries in Asian countries for the treatment of various diseases. Chung-Sang-Bo-Ha-Tang (CSBHT) contains medicinal herbs of 18 species and has been used in Korea for centuries for the treatment of chronic pulmonary diseases such as asthma^9^. However, since it is difficult to standardize the herbal formulation of CSBHT, the herbal preparation was modified to obtain the PM014 formulation, which comprises seven species of herbal extracts. We have demonstrated the potent anti-inflammatory effect of PM014 in lipopolysaccharide (LPS)-induced acute lung inflammation in murine models. *In vitro* and *in vivo* findings in another study demonstrated similar effects of PM014 in a murine chronic obstructive pulmonary disease (COPD) model^10^.

Stereotactic body radiotherapy (SBRT), a recently developed technique, delivers high doses of ablative radiation to tumours in a single fraction, with greater accuracy than conventional fractionated radiotherapy (CFRT). It has become the standard radiotherapy method for early-stage lung cancer^11^,^12^. However, there has been a lack of relevant mouse models for evaluating the effects of ablative radiation doses *in vivo*. In our previous study, we established an experimental radiation-induced lung inflammation mouse model using an image-guided animal irradiation method similar to SBRT^13^, for delivering a single dose of 75 Gy to the left lung of mice. The mice exhibited radiation pneumonitis at 2 weeks post-irradiation. In the present study, we investigated whether oral administration of PM014 reduces radiation-induced pneumonitis and influences lung function in an ablative radiation-induced lung inflammation mouse model.

## Results

### Gross Morphological Findings

**Figure 1:**
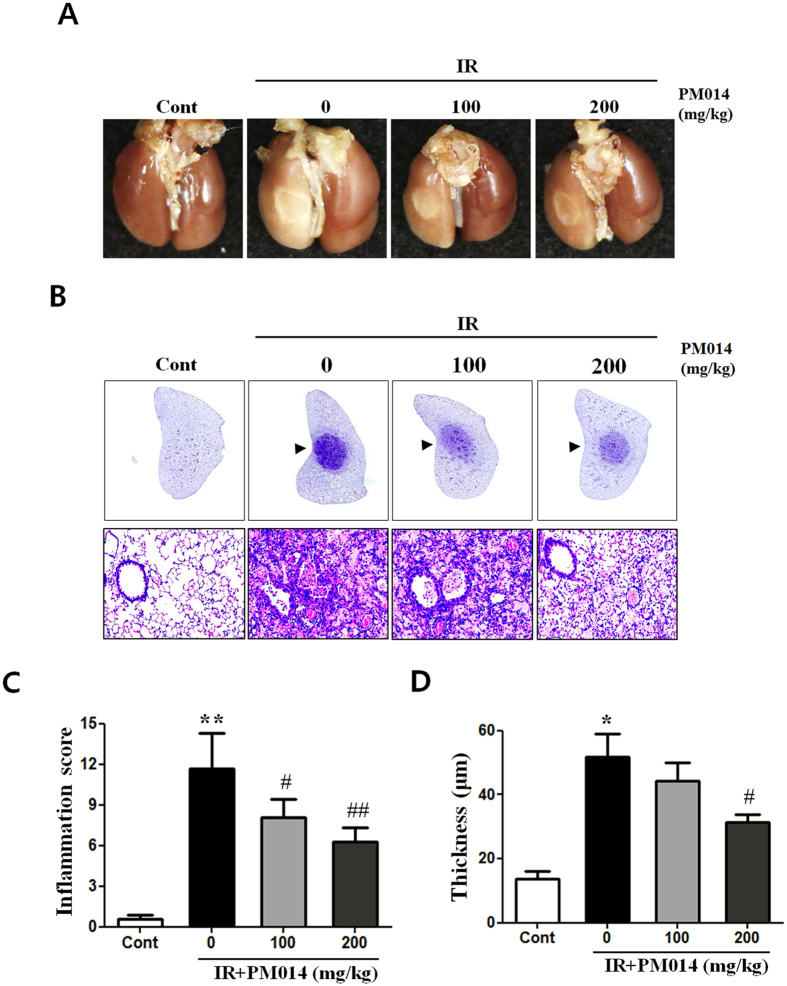
Effect of PM014 on gross morphology. Control, untreated; 0, irradiation (IR) +phosphate buffered saline (PBS); 100, irradiation +100 mg/kg PM014; and 200, irradiation +200 mg/kg PM014. (**A**) Mice were sacrificed at 2 weeks after irradiation. Lungs were photographed after complete fixation. (**B**) Haematoxylin and eosin-stained lung sections. Arrows indicate areas of radiation-induced injury. Magnification, 20x, 400x. (**C**) Quantification of inflammatory foci. (**D**) Thickness of bronchiolar epithelium. Data are expressed as mean ± standard error (*P < 0.05, **P < 0.01 versus control; ^#^P < 0.05 and ^##^P < 0.01, versus IR + PBS; n = 7–10).

To study the effect of radiation on lung morphology, we compared the morphological findings of lung tissue samples of irradiated and control mice. Irradiated areas in the left lung clearly exhibited local injury (Fig. 1A). In contrast to the brown colour of the lungs in control mice, the lungs of irradiated mice exhibited a definite, white, ring-like boundary, with white-coloured adjacent areas, indicating lung inflammation. PM014-treated mice (100 or 200 mg/kg) exhibited less inflammation than mice in the irradiation (IR) group.

### Effect of PM014 on Radiation-Induced Histological Changes in Lung Tissue

**Figure 2:**
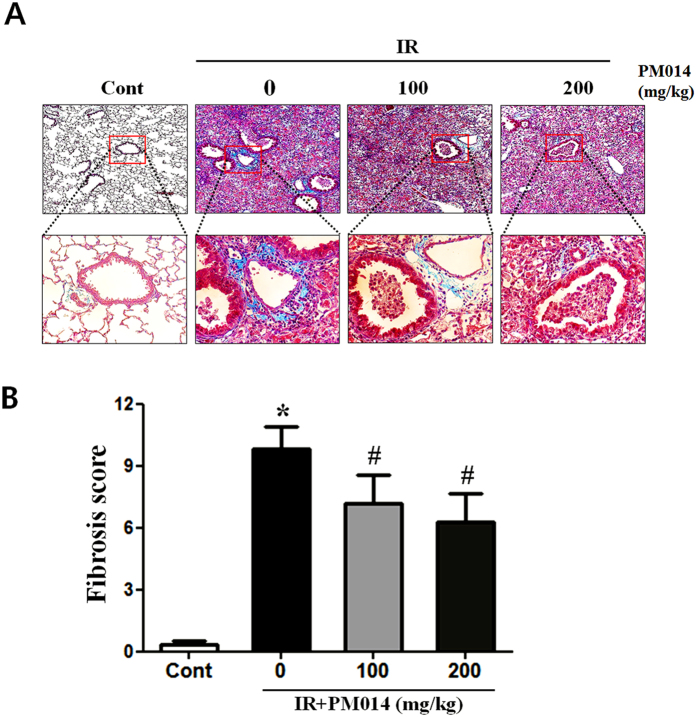
Effect of PM014 on radiation-induced lung fibrosis. Lung sections of all experimental groups at 2 weeks after irradation, stained with Masson’s trichrome stain: (**A**) Control, untreated; 0, irradiation (IR) +phosphate buffered saline (PBS); 100, irradiation +100 mg/kg PM014; and 200, irradiation +200 mg/kg PM014. Magnification, 100x, 400x. (**B**) Quantification of fibrotic foci. Data are expressed as mean ± standard error (*P < 0.05 versus control; ^#^P < 0.05 versus IR + PBS; n = 7–10).

Histopathological changes and inflammatory reactions due to radiation-induced lung damage in the lungs of irradiated mice included accumulation of numerous inflammatory cells in alveolar spaces and intra-alveolar hyaline membrane formation at 2 weeks post-irradiation. Alveolar infiltration of inflammatory cells in the IR group was significantly higher compared to that in the control group. PM014-treated mice exhibited reduced tissue damage, including lesser degrees of intra-alveolar hyaline membrane formation and inflammatory cell infiltration (Fig. 1B and C), and thickness of bronchiolar epithelium, which was measured from the base of columnar epithelium to the outer limit of the adventitia, supporting hyperplasia of the bronchiolar epithelium, in a dose-dependent manner (Fig. 1D). The results of Masson’s trichrome staining revealed that, in comparison with the control group, the IR group exhibited a marked increase in collagen deposition (Fig. 2). Treatment with PM014 (100 or 200 mg/kg) appears to have significantly reversed radiation-induced collagen deposition.

### Effect of PM014 on Inflammatory Cell Infiltration in the Lung Airway

**Figure 3:**
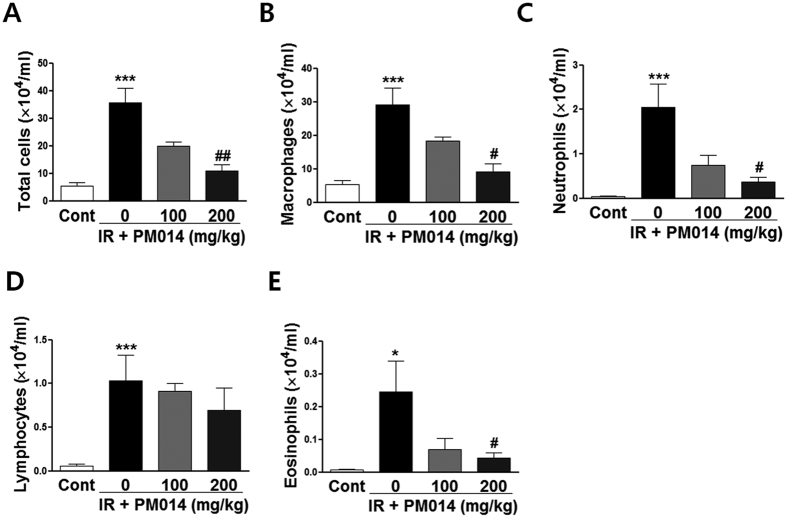
Effect of PM014 on infiltration of immune cells in bronchoalveolar lavage fluid (BALF) post-irradiation. Differential cell count of BALF at 2 weeks after irradation: total cell count (**A**), macrophages (**B**), neutrophils (**C**), lymphocytes (**D**), and eosinophils (**E**). Control, untreated; 0, irradiation (IR) +phosphate buffered saline (PBS); 100, irradiation +100 mg/kg PM014; and 200, irradiation +200 mg/kg PM014. Data are expressed as mean number of cells ± standard error (*P < 0.05 and ***P < 0.001 versus control; ^#^P < 0.05 and ^##^P < 0.01 versus IR + PBS; n = 7–10).

The total number of cells in BALF in the IR group was 6.55 times higher compared to that in the control group (Fig. 3A). In addition, the populations of macrophages (Fig. 3B), neutrophils (Fig. 3C), lymphocytes, (Fig. 3D) and eosinophils (Fig. 3E) in the IR group were significantly higher compared to those in the control group. In contrast, the populations of total cell, macrophages, neutrophils, and eosinophils in PM014-treated mice were lower compared to those in irradiated mice, with no significant changes in the number of lymphocytes.

### Effect of PM014 on Inflammation-Related Gene Expression in Lung Tissue

**Figure 4:**
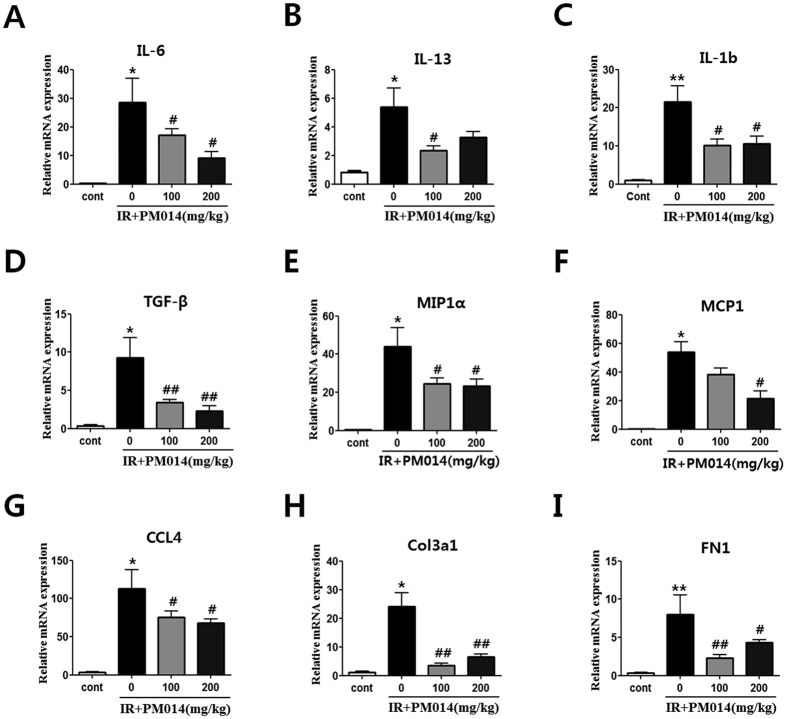
Effect of PM014 on mRNA levels. Control, untreated; 0, irradiation (IR) + phosphate buffered saline (PBS); 100, irradiation +100 mg/kg PM014; and 200, irradiation +200 mg/kg PM014. Data are expressed as mean number of cells ± standard error (*P < 0.05 and **P < 0.01 versus control; ^#^P < 0.05 and ^##^P < 0.01 versus IR + PBS; n = 7–10).

Expression of cytokines (IL-6, IL-13, IL-1b and TGF-β) (Fig. 4A–D), chemokines (MIP1a, MCP1, and CCL4) (Fig. 4E–G), and fibrosis-related (Col3al and Fn1) (Fig. 4H,I) genes was significantly increased in irradiated mice. Treatment with PM014 reduced expression of cytokines, chemokines and fibrosis-related genes.

**Figure 5:**
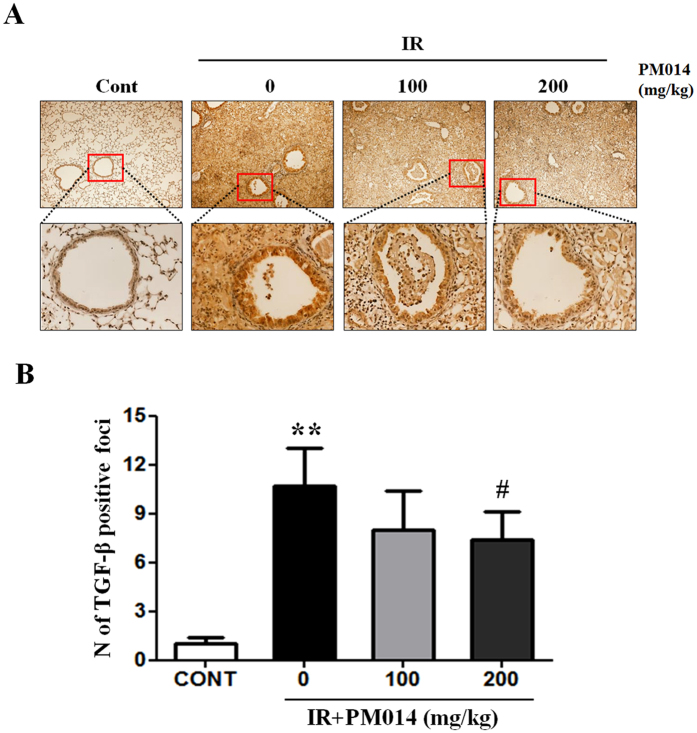
Effect of PM014 on TGF-β1 production in lung tissue. Immunohistochemistry images. Negative (score: 0–2) and positive (score: 3–9) TGF-β1 expression. Control, untreated; 0, irradiation (IR) + phosphate buffered saline (PBS); 100, irradiation +100 mg/kg PM014; and 200, irradiation +200 mg/kg PM014. Data are expressed as mean ± standard error (**P < 0.01 versus control and ^#^P < 0.05 versus IR + PBS; n = 7–10).

Additionally, we measured TGF-β1 protein levels in the lungs because TGF-β1 is an important pro-fibrotic growth factor that stimulates collagen synthesis in fibroblasts and myofibroblasts^14^. Immunohistochemical findings revealed high levels of TGF-β1 in irradiated mice. PM014 reduced TGF-β levels, in a dose-dependent manner (Fig. 5).

### Micro-Computed Tomography Findings

**Figure 6:**
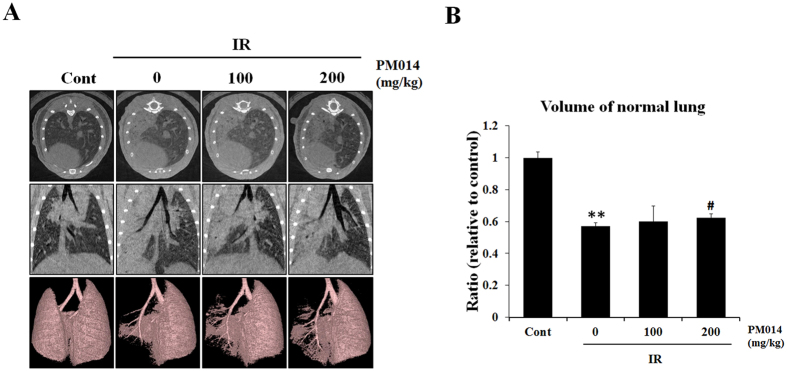
Micro-computed tomography (CT) findings. (**A**) Horizontal (top), trans-axial (middle), and 3D micro-CT (bottom) images acquired at 2 weeks after irradation. Control, untreated; 0, irradiation (IR) +phosphate buffered saline (PBS); 100, irradiation +100 mg/kg PM014; and 200, irradiation +200 mg/kg PM014. (**B**) Normal left lung volume (**P < 0.01 versus control; ^#^P < 0.05 versus IR + PBS).

Representative micro-CT images of the lungs of irradiated and control mice are presented in Fig. 6A. Two weeks after irradiation, pulmonary consolidation was observed throughout the left lung in irradiated mice; in contrast, PM014-treated mice exhibited fewer areas of consolidation. As shown in Fig. 6B, normal lung volume in the IR group was lower compared to that in control mice. However, normal lung volume appears to have significantly recovered in PM014-treated mice.

### Effect of PM014 on Functional Parameters of Lungs with Radiation-Induced Injuries

**Figure 7:**
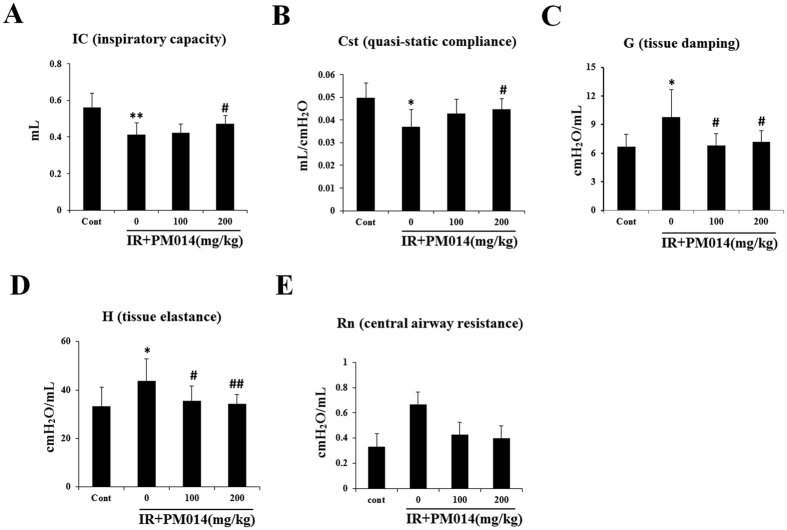
Effect of PM014 on functional parameters of the lungs in irradiated mice. Functional measurements of the mouse lung were collected with a flexivent system at 2 weeks after irradiation. Control, irradiated (IR), and IR + PM014 (100 and 200 mg/kg) groups. (*P < 0.05 and **P < 0.01 versus control; ^#^P < 0.05 and ^##^P < 0.01 vs. IR).

Functional lung parameters evaluated in this study are listed in Supplementary Table S2. There were significant differences in inspiratory capacity (IC), quasi-static compliance (Cst), tissue damping (G), and tissue elastance (H) of the lungs between IR group and control mice. The IC and Cst of the control group (0.56 ± 0.078 mL and 0.05 ± 0.006 mL/cm H_2_O, respectively) were significantly lower compared to those of the IR group (0.41 ± 0.062 mL [*P* < 0.01] and 0.037 ± 0.007 mL/cm H_2_O [*P* < 0.05], respectively). The values of G and H in the control group (6.69 ± 1.29 cm H_2_O/mL and 33.28 ± 7.77 cm H_2_O/mL, respectively) were higher compared to those in the IR group (9.81 ± 2.88 cm H_2_O/mL and 43.8 ± 9.06 cm H_2_O/mL, respectively) (Fig. 7). These results reflect the respiratory distress induced by irradiation in the IR group. However, radiation-induced respiratory distress in PM014-treated mice appears to have been significantly reversed. While mice treated with 100 mg/kg PM014 exhibited significant differences with IR group in only G and H values, mice treated with 200 mg/kg PM014 exhibited significant differences in all four parameters, indicating the protective effect of PM014 on radiation-induced lung injury. There were no significant differences in central airway resistance (Rn) between the control and IR groups or IR and PM014-treated groups.

## Discussion

Although radiotherapy is an effective treatment for lung cancer, application of curative doses of radiation might adversely affect healthy cells adjacent to tumours. Commonly, RILI involves early inflammatory response (pneumonitis) and late pulmonary fibrosis. Radiation pneumonitis, in particular, can cause a significant morbidity and mortality^15^,^16^. In the present study, we investigated whether PM014, a herbal mixture, has any influence on radiation-induced lung inflammation using an ablative radiation-induced acute lung injury mouse model.

Radiation-induced lung injury occurs in up to 30% of patients with lung tumours and 10–15% of those with other thoracic cancers, who receive radiotherapy^17^. In recent times, SBRT has been used widely for cancer treatment, because it delivers higher radiation doses with greater accuracy than CFRT. Several recent studies on SBRT for lung cancer have reported superior treatment outcomes^11^,^12^. However, other studies have reported that high-dose radiotherapy results in more severe damage to normal tissues, such as stromal tissues, including vasculature, than CFRT^18^,^19^, which highlights the need for appropriate *in vivo* models for evaluation of adverse effects of SBRT. Therefore, we developed an *in vivo* model using an image-guided, high-focus irradiation system similar to SBRT^20^.

In traditional Korean medicine, CSBHT is well-known as a herbal mixture for treatment of pulmonary diseases. Previously, we had compared the effects of PM014, which comprises seven major components of CSBHT^9^, with those of its individual constituent herbs in an acute LPS-induced lung injury model. PM014 treatment resulted in a greater reduction of immune cell infiltration in the lungs than treatment with individual herbs^10^. To find the optimal dosage of PM014 on X-ray induced mouse lung injury model, we did preliminary experiment with 50, 100, 200 and 300 mg/kg doses of PM014. As a result, a dose of 200 mg/kg of PM014 is likely the most efficient concentration needed to elicit the inhibitory effects on radiation-induced lung inflammation. When we evaluated the doses used in this study, compared with the doses that were administered therapeutically, we referred to the guidance for industry prepared by the Office of New Drugs in the Center for Drug Evaluation and Research at the Food and Drug Administration^21^. According to the guidance, when we treated mice with 200 mg/kg of PM014, 1 g of PM014 is an equivalent dose for 60 kg humans. To find the optimal numbers of administration of PM014 on X-ray induced inflammation, we did a preliminary experiment with single, once a week (2 times for 2 weeks), and continual treatment of PM014 (6 times for 2 weeks). As a result, single and once a week treatment did not show significant effects on radiation-induced inflammation. Therefore, we decided to use continual treatment of PM014 for the entire time duration following irradiation.

Irradiation causes tissue fibrosis, which is characterized by excessive accumulation of collagen and extracellular matrix (ECM) as well as remodelling of lung architecture, thus leading to decreased organ function^22^,^23^. In the present study, we evaluated the effects of PM014 on tissue fibrosis—in terms of collagen and ECM accumulation as well as remodelling of lung architecture—and inflammatory response—in terms of immune cell infiltration and cytokine expression—at 2 weeks post-irradiation. Tissue fibrosis starts as acute interstitial inflammation at 6–12 weeks post-irradiation, progresses to productive chronic inflammation lasting several months, and culminates in lung fibrosis and scar formation several months to years after radiation therapy^24^,^25^. In the present study, collagen accumulation was observed at 2 weeks after irradiation. PM014 treatment resulted in the marked decrease of not only collagen accumulation around bronchioles but also immune cell infiltration (Figs 2 and 3). It also inhibited the thickening of epithelial basement membrane and bronchiole inflammation. Bronchiolar epithelium thickening may occur due to inflammation^26^, which was measured from the base of columnar epithelium to the outer limit of the adventitia supporting hyperplasia of the bronchiolar epithelium. Airway epithelium represents the first line of defense against toxic environments^27^. The airway epithelium is composed of multiple different cell types, which produce and release mucous into the apical surface of the epithelium thus trapping foreign particles^28^. However, over-production of mucous or hyperplasia of mucous-producing cell in the airway epithelium can have deleterious effects by creating mucous plugs and thus leading to airway obstruction^29^.

During inflammation, inflammatory cells such as macrophages, lymphocytes, neutrophils, and fibroblasts are activated to release inflammatory and profibrotic cytokines^6^. Macrophages are of two subtypes with distinct functions — classic M1-activated macrophages (M1) and alternatively activated macrophages (M2)^30^. M2 macrophages, which are associated with radiation-induced fibrotic processes^31^, express TGF-β1, arginase 1, and PDGF, which stimulate myofibroblast differentiation and production of ECM proteins^30^,^32^. Our results indicated that treatment with 200 mg/kg PM014 significantly reduced inflammatory cell infiltration in BALF (Fig. 4) and brought about a marked reduction in the macrophage population. These results suggest that the anti-fibrotic effects of PM014 may be largely attributed to the suppression of macrophage activation, especially that of the M2 type. However, further experiments regarding M1/M2 macrophage polarization by PM014 are necessary to confirm these effects.

Pro-inflammatory and pro-fibrotic cytokines such as TGF-β, TNF-α, IL-6, and IL-13 contribute to the pathogenesis of tissue fibrosis^22^,^33^,^34^,^35^. Transforming growth factor-β, a multifunctional cytokine produced by neutrophils, macrophages, and fibroblasts, plays a central role in matrix production, epithelial cell apoptosis, epithelial-to-mesenchymal transition, wound healing, and tissue remodelling after injury^36^,^37^,^38^,^39^,^40^. It also plays an immunomodulatory role in the regulation of lung fibrosis through recruitment and activation of fibroblasts and immune cells^5^. Chemokines such as MIP1a, MCP1, and CCL4 act predominantly as chemoattractants for monocytes and lymphocytes to the site of lung injury^41^. Our findings indicate that, while irradiation significantly increased the expression of cytokines (IL-6, IL-13, IL-1b and TGF-β), pro-fibrotic factors (Col3a1 and Fn1), and chemokines (MIP1a, MCP1 and CCL4), PM014 treatment decreased not only the expression of most of these proteins (Fig. 5), but also inflammatory cell recruitment and fibrosis of lung tissue.

In our previous report, we had reported that radiation-induced lung inflammation was correlated with inflammasome activation^42^. Inflammasomes play a key role in acute and chronic immune responses to radiation and control the production of important pro-inflammatory cytokines^43^,^44^. Thus, regulation of inflammasome activation may reduce radiation-induced inflammation. To evaluate potential mechanisms of PM014 in the treatment of radiation induced lung inflammation, we performed real-time PCR. Consistent with our previous report, the expression of inflammasome-related genes, *Nlrp3, Il1a, Il-1b*, and *Casp1*, increased in irradiated mice. However, treatment with PM014 reduced expression of these inflammasome-related genes (Supplementary Figure S1), suggesting that PM014 may suppress radiation-induced inflammasome activation pathways. Thus, we are currently studying possible signaling pathway triggers for inflammasome activation following radiation.

Micro-CT is used for the detection and quantitative analysis of early structural and histopathological changes associated with lung injury induced by ablative doses of focal volume radiation. It has the ability to not only better delineate parenchymal changes, but also demonstrate changes restricted to the irradiated field, thus making diagnosis easy and accurate. The results of micro-CT analysis in the present study correlated with the histopathologic findings. Characteristic CT features of SBRT-induced lung injuries include ground-glass opacity and consolidation, which were observed at 2 weeks post-irradiation in irradiated mice in the present study. PM014 treatment appears to have resulted in the partial resolution of these features (Fig. 7). Herbs of seven species in PM014 were also evaluated in regards to their therapeutic effects. Among individual herbs, Fruit of *Schizandra chinensis* and Root of *Scutellaria baicalensis* showed some attenuation effects on X-ray induced lung inflammation in the mice (Supplementary Figure S2), compared to other herbs, which were reported to exhibit medicinal effects such as renal protective and anti-inflammatory effects, respectively^36^,^45^,^46^. Although the therapeutic effective components of PM014 have not yet been fully characterized, we could suggest that PM014 was the most ef-6fective therapeutic agent, compared to the individual herbs, and that PM014 is likely to achieve a more helpful effect based on the synergy of its individual compontents.

FlexiVent^TM^ is an achievable measurement system with a pre-programmed ventilator, which directly evaluates lung function based on the same functional parameters as those used in humans^47^. In the present study, PM014-treated mice exhibited significantly better values of lung function parameters including IC, Cst, G, and H than irradiated mice, which suggests that PM014 treatment improves lung function.

Taken together, our results suggest that PM014 reduces radiation-induced lung inflammation and may, therefore, be used as a therapeutic agent for the inhibition of radiotherapy-induced inflammatory response and reversal of early fibrosis in normal lung tissue.

## Methods

### Animal Experiments

All protocols involving the use of mice were approved by the Animal Care and Use Committees of the Kyung Hee University (KHUASP(SE)-15-020) and Yonsei University Medical School (2014-0164-1) and were performed in accordance with the relevant guidelines. Female C57BL/6 mice (age, 6 weeks; weight, 20–25 g) were purchased from Charles River Korea (Orient Bio, Seongnam, South Korea) and allowed to acclimatize (n = 5 per cage) for a week before irradiation. A single dose of 75 Gy was delivered to the left lung in a single fraction using an image-guided small-animal irradiator (X-RAD 320; Precision, North Branford, CT, USA) equipped with a collimator system composed of 3.5-cm-thick copper to produce focal radiation beams as well as an imaging subsystem consisting of a fluorescent screen coupled to a charge-coupled-device camera. We selected 3-mm collimators to mimic clinical SBRT conditions by irradiating only a small volume of tissue. The mice were randomly divided into four groups (n = 6–8 per group) as follows: (1) control group — mice were orally administered phosphate buffered saline (PBS) on days 3, 5, 7, 9, 10, and 13; (2) irradiation (IR) + PBS group — mice were exposed to a single dose of 75 Gy delivered to the left lung in a single fraction and orally administered PBS on days 3, 5, 7, 9, 10, and 13; and (3) two groups of PM014-treated mice according to the dosage of PM014 (100 mg/kg or 200 mg/kg) mice were orally administered PM014 on every other day after irradiation. On day 14, the mice were sacrificed by CO_2_ asphyxiation, and lung tissues were collected for analysis.

### Preparation of PM014

Medicinal plants of the seven species that constitute PM014 were purchased from Kyung Hee Herb Pharm (Seoul, South Korea) and processed at Hanlim Pharm Co. LTD (Seoul, South Korea). The herbs were cut and mixed to a total weight of 2100 g according to the ratio presented in Supplementary Table S1. The mixture was extracted with purified water (2100 mL) using a reflux condenser for 3 h at 90–100 °C and then filtered using a 25-μm sieve. The supernatant was concentrated at 60 °C under vacuum, using an evaporator. The extracts were mixed with 260 g dry cornstarch and vacuum dried at 60 °C. For administration, the PM014 extract was dissolved in PBS. The quantities of standard materials in 1 g of the final PM014 extract were: Paeoniflorin >0.43 mg, Schizandrin >0.12 mg, Baicalin >7.26 mg, and Amygdalin >2.48 mg. Quantification of standard materials in PM014 was performed by high-performance liquid chromatography analysis. Three independent batches of each compound were analysed for obtaining triplicate data. The standardized herbal formula of PM014 has been approved for the Investigational New Drug (IND) program by the Ministry of Food and Drug Safety, Republic of Korea (ID: 20130030575).

### Analysis of Bronchoalveolar Lavage Fluid

After sacrifice, a 1-mL syringe containing 1 mL PBS was inserted into the exposed trachea of mice. The PBS was injected and aspirated back into the syringe to collect bronchoalveolar lavage fluid (BALF). This procedure was repeated three times. The fluid was centrifuged at 1300 rpm for 10 min. Cell pellets were resuspended in 1 mL PBS and collected on glass slides by cytocentrifugation. Total live-cell count was determined using a haemocytometer. In addition, the cell counts of macrophages, neutrophils, eosinophils, and lymphocytes were determined by evaluating BALF cytospin smears stained with the Diff-Quick stain (Life Technologies, Auckland, New Zealand); 500 cells were counted per slide. The supernatants of BALF were stored at −80 °C for further analysis.

### Preparation of lung tissues for histology and immunohistochemistry

Left-lung tissues of irradiated mice were fixed in 4% paraformaldehyde and then dehydrated and embedded in paraffin. For histological study, 4 μm tissue sections were stained with haematoxylin and eosin (H&E), Masson’s trichrome (MT) and immunohistochemical (IHC) stains. For detection of TGF-β1, tissue sections were incubated with an anti-TGF-β1 primary antibody (1:100 dilution; ab64715, Abcam) at 4 °C overnight. Slides were then incubated with avidin–biotin peroxidase complex (ABC kit, Vector Laboratories, CA, USA) and were developed using 3,3′-diaminobenzidine tetrachloride (DAB; Zymed Laboratories, CA, USA).

### Histology and immunohistochemistry evaluation

Slides were assessed according to a dual-rate semi-quantitative method by three independent pathologists, who were blinded to sample identities^48^. For histological evaluation, lung tissue sections were stained with H&E and MT staining and scored for the number of inflammation or fibrotic foci, respectively. For IHC evaluation, lung tissue sections were stained with TGF-β1 staining. Randomly selected fields of each slide were scored for area and intensity of positively stained (brown) cytoplasm and cell membrane. Intensity scores were assigned as follows: 0 = no appreciable staining (negative); 1 = barely detectable staining (weak); 2 = readily appreciable brown staining (moderate); and 3 = dark brown staining (strong positivity). The total score was calculated by adding the intensity scores from five independent views in each sample, resulting in a final score of 0 to 15. For statistical analysis, scores 3–15 and 0–2 were defined as indicating positive and negative expression, respectively.

### Micro-Computed Tomographic Analysis

Micro-computed tomography (CT) images were acquired using a volumetric CT scanner (NFRPolaris- G90MVC: NanoFocusRay, Iksan, South Korea) at 50 kVp, 180 μA, and 150 mGy (number of views, 700; frame rate, 142 ms). Images were reconstructed (image size, 1232 × 1120 pixels; number of slices, 512) by volumetric cone-beam reconstruction (Feldkamp-Davis-Kress method) in in-line/off-line modes. Volumetric analysis was performed using the Image J software. In order to minimize inter-specimen variations in measurement, identical level settings were used for analysis of all images.

### Functional assessment of the lungs

Lung function in irradiated mice was evaluated with the Flexivent system (Flexivent^®^; SCIREQ©, Montreal, QC, Canada), which measures flow-volume relationships in the respiratory system, including forced oscillation, to discriminate between airway and lung tissue variables (A)^13^. Evaluations were performed according to the manufacturer’s instructions.

Briefly, after anesthetization, mice were connected to a computer-controlled small-animal ventilator and quasi-sinusoidally ventilated with a tidal volume of 10 mL/kg at a frequency of 150 breaths/minute. Measurement commenced when a stable ventilation pattern without obvious spontaneous ventilator effort was observed at the ventilation pressure tracing. All perturbations were performed sequentially until three acceptable measurements (coefficient of determination >0.95) were recorded for each subject, from which an average was calculated.

### Real-time Reverse Transcription-Polymerase Chain Reaction

RNA was isolated from lung tissues using the RNeasy Mini Kit (Qiagen, CA, USA) according to the manufacturer’s instructions and quantified using a spectrophotometer (NanoDrop; ND-1000; NanoDrop Technologies, Inc., Wilmington, DE, USA). Real-time reverse transcription-polymerase chain reaction (RT-PCR) was performed to quantitate the expression of IL-6, IL-13, IL-1b, CCL-4, collagen type III alpha 1 (COL3a1), fibronectin (FN1), macrophage inflammatory protein (MIP)-1a, and monocyte chemotactic protein (MCP)-1 using the Light Cycler 480 SYBR Green I master mix and Light Cycler 480 real-time PCR machine (Roche Applied Science, Indianapolis, IN, USA). Quantification was performed by the comparative CT method (ΔΔCT). Data were obtained from three independent PCR experiments and are represented as mean ± standard error (SE).

The following mouse primer sequences were used for amplification: *Il-6* (F 5′-ccggagaggagacttcacag-3′; R 5′-tccacgatttcccagagaac-3′); *Il-13* (F 5′-cagcatggtatggagtgtgg; R 5′-aggccatgcaatatcctctg); *Il-1b* (F 5′-gcccatcctctgtgactcat-3′; R 5′-aggccacaggtattttgtcg-3′); *Ccl4* (F 5′-cccacttcctgctgtttctc-3′; R 5′-gtctgcctcttttggtcagg-3′); *Col3a1* (F 5′-accaaaaggtgatgctggac-3′; R 5′-gacctcgtgctccagttagc-3′); *Fn1* (F 5′-acagagctcaacctccctga-3′; R 5′-tgtgctctcctggttctcct-3′); *Mip1a* (F 5′-atgaaggtctccaccactgc-3′; R 5′-gatgaattggcgtggaatct-3′); *Mcp1* (F 5′-ccaatgagtaggctggaga-3′; R 5′-tctggacccattccttcttg-3′); *TGF-β* (F 5′-agcggactactatgctaaagaggtcaccc-3′; R 5′-ccaaggtaacgccaggaattgttgctata-3′); and *β-actin* (F 5′-gatctggcaccacaccttct-3′; R 5′-ggggtgttgaaggtctcaaa-3′).

### Statistical Analysis

Statistical analysis was performed using the Prism 5 software (Graph Pad Software Inc., San Diego, CA, USA). Comparison of variables between the control and radiation-treatment groups was performed by Mann-Whitney U test. Values of *p* < 0.05 were considered statistically significant.

## Additional Information

**How to cite this article:** Kim, J.-Y. *et al*. Standardized Herbal Formula PM014 Inhibits Radiation-Induced Pulmonary Inflammation in Mice. *Sci. Rep.* **7**, 45001; doi: 10.1038/srep45001 (2017).

**Publisher's note:** Springer Nature remains neutral with regard to jurisdictional claims in published maps and institutional affiliations.

## Supplementary Material

Supplementary Information
