# Supplementary material for: Standardized Herbal Formula PM014 Inhibits Radiation-Induced Pulmonary Inflammation in Mice
Source: Sci Rep. 2017 Mar 21;7:45001. doi: 10.1038/srep45001 (PMC5359558; doi:10.1038/srep45001)
Supplement: Supplementary Information [file srep45001-s1.pdf]

Supplementary Information

Title : Standardized Herbal Formula PM014 Inhibits Radiation-Induced Pulmonary Inflammation in Mice

Jee-Youn Kim[1,†], Dasom Shin[2,†], Gihyun Lee[2], Jin-Mo Kim[1], Dongwook Kim[2], Yong-Min An[1], Byung Rok Yoo[1], Hanna Chang[1], Miran Kim[3], Jaeho Cho[1,*] & Hyunsu Bae[2,*]

Supplementary Table S1. Composition and amount of PM014

Formula of PM014	Amount (g)	Chemical marker
Root of *Rehmannia glutinosa* (RG)	600	5-HMF
Cortex of *Paeonia suffruticosa* (PS)	300	Paeoniflorin
Fruit of *Schizandra chinensis* (SC)	300	Schizandrin
Root of *Asparagus cochinchinensis* (AC)	300	Asparagine
Seed of *Prunus armeniaca* (PA)	225	Amygdalin
Root of *Scutellaria baicalensis* (SB)	225	Baicalin
Root of *Stemona sessilifolia* (SS)	150	Stemonine
Total	2,100 g	

Supplementary Table S2

The parameter description using the flexiVent™ system measurements

Parameter	Abbreviations	Descritption
Inspiratory Capacity	IC	IC is the volume difference between functional residual capacity (FRC) and total lung capacity (TLC), Also equaling tidal volume plus the inspiratory reserve volume
Quasi-static Compliance	Cst	The parameter K of the Salazar-Knowles equation reflects the curvature of the upper portion
Tissue Damping	G	Tissue damping is closely related to tissue resistance and reflects the energy dissipation in the lung tissues.
Tissue Elastance	H	The parameter H is closely related to tissue elastance and reflects the energy conservation in the lung tissues.
Newtonian Resistance	Rn	The Newtonian Resistance parameter of the Constant Phase Model represents the resistance of the central airway.
Airway constriction	Rrs	Resistance of the respiratory system
Forced Expiration Volume	FEV	Amount of air forcefully exhaled (drawn) at different time points
Forced Vital Capacity	FVC	Amount of air the animal can physically exhale

Supplementary Figure S1

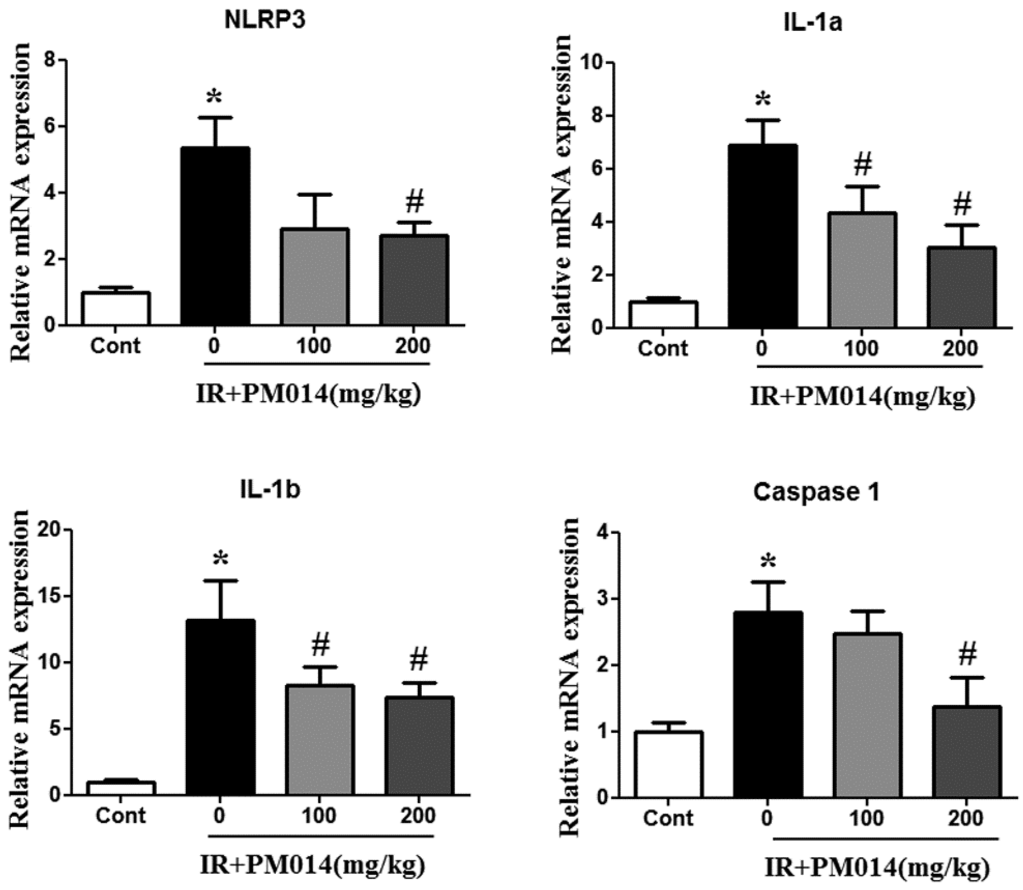

Supplementary Figure S2

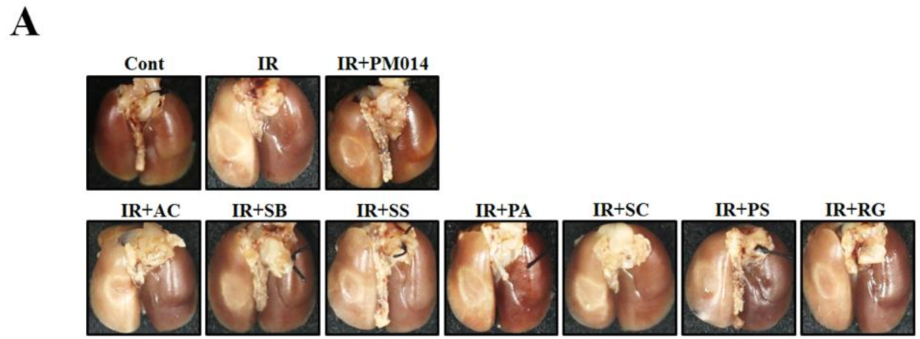

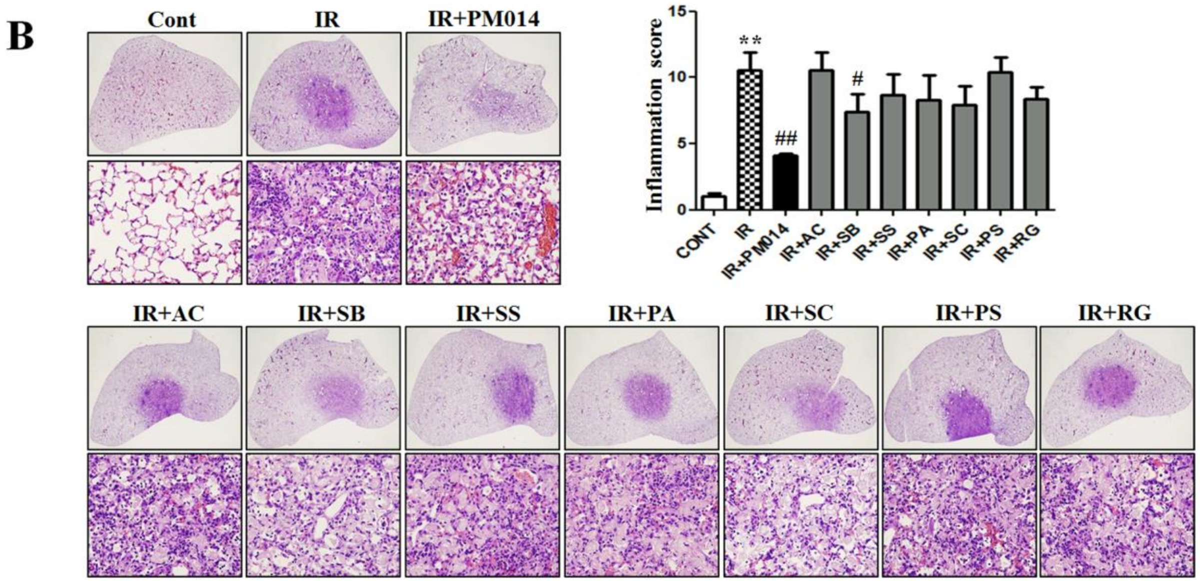

C

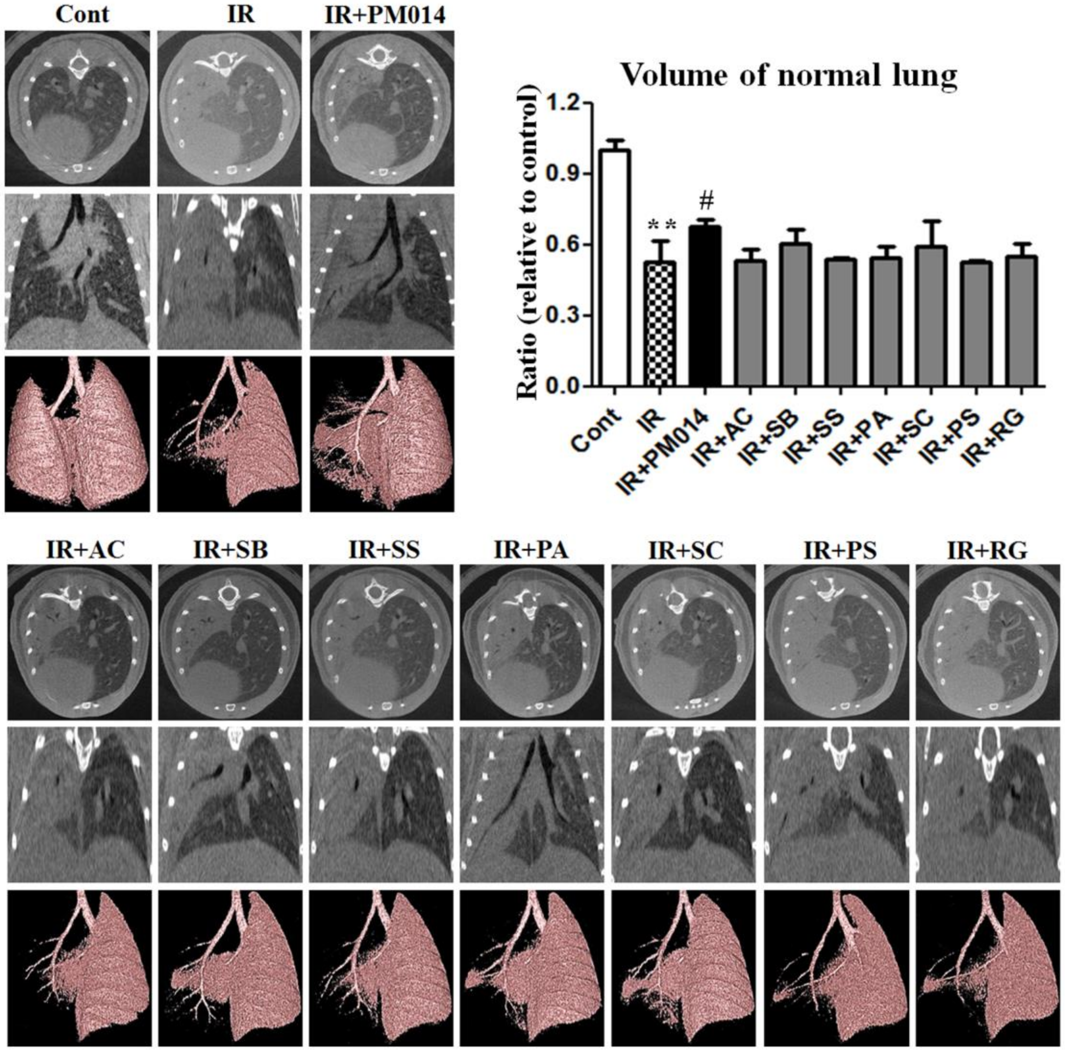
Cont
IR
IR+PM014
Volume of normal lung
Ratio (relative to control)
1.2
0.9
0.6
0.3
0.0
**
#
Cont
IR
IR+PM014
IR+AC
IR+SB
IR+SS
IR+PA
IR+SC
IR+PS
IR+RG
IR+AC
IR+SB
IR+SS
IR+PA
IR+SC
IR+PS
IR+RG

Supplementary Figure S1. Effect of PM014 on mRNA of inflammasome-related genes after 75Gy irradiation.

Quantitative real-time polymerase chain reaction (RT- PCR) analysis showed that PM014 treatment decreased mRNA expression of inflammasome-related genes in the mouse lungs. cDNA was synthesized from the total RNAs extracted from irradiated mouse and subjected to RT-PCR analysis. Control, untreated; 0, irradiation (IR); 100, irradiation + 100 mg/kg PM014; and 200, irradiation + 200 mg/kg PM014. Data are expressed as mean number of cells ± standard error (*P < 0.05 versus control; $^{\#}$P < 0.05 versus IR). The sequences of the mouse primers were as follows: *Nlrp3* (FW 5'-atgctgcttcgacatctcct-3'; RW 5'-gtttctggaggttgcagagc-3'), *Il1a* (FW 5'-ccgacctcattttcttctgg-3'; RW 5'-gtgcacccgactttgttctt-3'), *Il1b*(FW 5'-gcccatcctctgtgactcat-3'; RW 5'-aggccacaggtattttgtcg-3'), *Casp1* (FW 5'-cacagctctggagatggtga-3'; RW 5'-ggtcccacatattccctcct-3').

Supplementary Figure S2. Effect of individual herbs in PM014 on gross morphology, histological and micro-CT analysis at 2 weeks after 75 Gy irradiation

Mice were exposed to a single dose of 75 Gy delivered to the left lung in a single fraction and orally administered PM014 (200 mg/kg) or each herb extract (in proportion to PM014) on days 3, 5, 7, 9, 10, and 13, and then sacrificed at 2 weeks after irradiation. (A) Lungs were photographed after complete fixation. (B) Haematoxylin and eosin-stained lung sections and quantification of inflammatory foci. (C) micro-CT findings. Horizontal (top), trans-axial (middle), and 3D micro-CT (bottom) images and quantification of volume of normal lung. Control, untreated; IR, irradiation; AC, Root of *Asparagus cochinchinensis*; SB, Root of *Scutellaria baicalensis*; SS, Root of *Stemona sessilifolia*; PA, *Seed of Prunus armeniaca*; SC, Fruit of *Schizandra chinensis*; PS, Cortex of *Paeonia suffruticos*; RG, Root of *Rehmannia glutinosa*. (**P < 0.01 versus control; $^{\#}$P < 0.05 and ##P < 0.01 versus IR).
